# Expanding the Spectrum of Canine Diffuse Large B‐Cell Lymphoma Genetic Aberrations Through Whole Genome Sequencing Analysis

**DOI:** 10.1111/vco.13059

**Published:** 2025-05-04

**Authors:** Antonella Fanelli, Eugenio Mazzone, Diana Giannuzzi, Laura Marconato, Luca Aresu

**Affiliations:** ^1^ Department of Veterinary Sciences University of Turin Grugliasco Turin Italy; ^2^ Department of Agronomy, Food, Natural Resources, Animals and Environment (DAFNAE) University of Padua Legnaro Padua Italy; ^3^ Department of Veterinary Medical Sciences University of Bologna Ozzano dell'Emilia Bologna Italy

**Keywords:** diffuse large B‐cell lymphoma, DLBCL, dog, prognosis, whole genome sequencing

## Abstract

Diffuse large B‐cell lymphoma (DLBCL) is one of the most prevalent haematological malignancies in both humans and dogs, characterised in both species by significant clinical heterogeneity and limited prognostic predictability. With the introduction of next‐generation sequencing (NGS) technologies in veterinary medicine over the past decade, researchers have begun to elucidate the molecular basis of canine DLBCL (cDLBCL); however, much of the clinical heterogeneity associated with this tumour remains unexplained. In this study, we performed whole genome sequencing on 10 cDLBCL cases, all treated with chemo‐immunotherapy, which exhibited similar clinico‐pathological features but markedly different outcomes. Cases were classified as “poor” or “good” responders based on whether their lymphoma‐specific survival fell below or above the cohort's median. Protein‐coding variants and copy number aberrations unique to poor or good responders revealed novel candidate genes not previously identified in cDLBCL studies, while splicing, untranslated regions, and intronic variants were detected in genes already known to be recurrently mutated. In conclusion, our investigation has broadened the spectrum of potentially pathogenic variants implicated in cDLBCL, though further studies with larger cohorts are necessary to validate these findings.

## 1 Introduction

Diffuse large B‐cell lymphoma (DLBCL) is the most prevalent and deadly hematologic malignancy in dogs, with clinical manifestations that are highly variable and not reliably predictable based on presentation alone [1]. Recent advances in molecular profiling have shed light on the complex landscape of canine DLBCL (cDLBCL) through comprehensive transcriptomic [2, 3], methylome [4], and genomic analyses, utilising both whole exome [5, 6, 7] and targeted sequencing approaches [8]. These investigations have uncovered distinctive gene expression patterns, copy number aberrations (CNAs), and recurrently mutated genes that drive the pathogenesis of cDLBCL and help stratify patients into prognostically distinct subgroups. Notably, the transcriptional profile of cDLBCL closely mirrors the human activated B‐cell (ABC) subtype of DLBCL, which is characterised by unique signatures associated with the activation of the B‐cell receptor (BCR) and nuclear factor‐κB (NF‐κB) pathways [3].

Notably, *TP53* mutations have emerged as strong independent predictors of poor prognosis, while dogs with wild‐type *TP53* show significant therapeutic benefit from the addition of immunotherapy to standard CHOP‐based protocols [7]. These findings strongly advocate for routine *TP53* mutational screening during the initial clinical assessment to aid in both prognostic stratification and informed therapeutic decision‐making. Despite advances in understanding the molecular pathogenesis of cDLBCL, substantial clinical and prognostic heterogeneity remains unexplained. While gene expression and mutational profiles have been extensively characterised, the non‐coding genome of cDLBCL remains largely unexplored.

In human oncology, the historically high costs of whole genome sequencing (WGS) have led to a preference for whole exome sequencing (WES) or targeted gene panels. Consequently, most available data have focused on the coding regions of the genome, concentrating on clinically relevant genes. This focus has left the potential contributions of untranslated, intronic, and intergenic regions to tumour development and progression largely unexplored [9]. The discovery of activating *TERT* promoter mutations across multiple human cancer histotypes [10] and the identification of numerous cancer susceptibility *loci* within non‐coding regions through genome‐wide association studies (GWAS) [11, 12] have highlighted the potential of non‐coding variants as significant drivers of tumorigenesis. These findings, alongside the decreasing costs of WGS, have catalysed a substantial increase in the publication of tumour genomes, culminating in the Pan‐Cancer Analysis of Whole Genomes (PCAWG) project. This collaborative effort between The Cancer Genome Atlas (TCGA) and the International Cancer Genome Consortium (ICGC) has analysed over 2500 cancer genomes, uncovering novel mutational signatures, structural variants, and insights into tumour evolution [13, 14].

In dogs, to date, only a single study has employed WGS to analyse six cases of multicentric B‐cell lymphoma (BCL) [15]. However, this investigation was limited in scope, focusing exclusively on protein‐coding gene variants and relying on a generic BCL diagnosis without histotype specification or correlation with clinical data. Additionally, the use of potentially contaminated blood samples as matched‐normal DNA controls, without flow cytometric confirmation to rule out neoplastic infiltration, may have compromised the comparative analyses, further limiting the study's findings.

The present study aims to provide a comprehensive characterisation of the genome‐wide mutational landscape of cDLBCL to elucidate the complex molecular mechanisms driving its pathogenesis. To facilitate this investigation, we selected 10 cases with similar clinico‐pathological features but divergent survival outcomes.

## 2 Methods

### 2.1 Samples

Ten cDLBCL from client‐owned dogs were selected from the archive of the Canine Lymphoma Biobank [16]. The study did not fall within the application areas of Italian Legislative Decree 26/2014 which governs the protection of animals used for scientific or educational purposes; therefore, ethical approval was waived for this study by the Department of Veterinary Sciences at the University of Turin. All methods were carried out in accordance with relevant guidelines and regulations, and dog owners gave written informed consent.

To be included in the analysis, dogs had to be treated with chemoimmunotherapy consisting of the administration of the APAVAC vaccine in addition to a standardised CHOP‐based protocol as previously described [17]. The following patient demographics and clinico‐pathological features were available: clinical stage, substage, immunophenotype determined by flow cytometry on a neoplastic lymph node aspirate, infiltration of peripheral blood (PB) and bone marrow (BM) determined by flow cytometry, serum lactate dehydrogenase (LDH) activity and whether the dogs had been pre‐treated with steroids. For stage V disease, the cutoff for PB infiltration was > 1%, even in the absence of BM involvement (BM infiltration > 3%). All dogs underwent lymphadenectomy for routine histologic analysis and immunohistochemistry (CD3 and CD20), vaccine preparation, and DNA extraction. Time to progression (TTP), lymphoma‐specific survival (LSS) and cause of death were also available. TTP was measured as the time between the start of treatment and disease progression, while LSS was calculated from the start of treatment to death due to lymphoma.

### 2.2 DNA Isolation and Sequencing

Total DNA was extracted from RNA‐later preserved tumour samples and skin punch biopsies as matched‐normal tissues, as they appeared macroscopically and histologically normal. Since cutaneous infiltration by lymphoma typically progresses into visible lesions, its absence in all included dogs suggested no apparent skin involvement. The AllPrep DNA/RNA Mini Kit (Qiagen, Hilden, Germany) was used according to the manufacturer's instructions. DNA concentration and quality were assessed by Qubit fluorometer (Life Technologies Ltd., Paisley, UK) and agarose gel electrophoresis. Twenty high‐quality WGS libraries were prepared using the Illumina‐compatible KAPA HyperPlus Library Preparation Kit (Roche Sequencing and Life Science, Wilmington, MA). Libraries were quantified through a Qubit 2.0 Fluorometer using the Qubit DNA Assay Kit (Thermo Fischer, Foster City, CA, USA) and quality was assessed using the Bioanalyzer 2100 instrument (Agilent Technologies, Santa Clara, CA, USA). Ten libraries (fragments ranging from 300 to 400 bp) were then pooled and sequenced on an Illumina NovaSeq 6000 platform in a paired‐end (150 PE) mode with an average coverage of 30×. Raw Illumina sequencing data are deposited in BioProject database with BioProject ID PRJNA805123. The data will be available following an embargo from the date of publication.

### 2.3 WGS Data Pre‐Processing

Firstly, all raw sequencing data underwent a quality check using fastqc software to ensure that no errors occurred during the preprocessing step. They were then processed according to the Genome Analysis Toolkit (GATK) preprocessing pipeline, which is the gold standard in human and veterinary medicine. Briefly, reads were aligned to the reference genome (canFam3.1, UCSC) using Burrows‐Wheeler Aligner (BWA), converted to bam format (Samtools), sorted by coordinate (Samtools), read groups were added (Picard) and duplicates were marked (Picard). Finally, all data underwent Base Quality Score Recalibration.

### 2.4 Somatic Variants Calling

Somatic variant calling was performed to access single nucleotide variants (SNVs) and small insertion/deletion variants (indels). First, matched‐normal tissue sequencing data were elaborated using Mutect to collect Single Nucleotide Polymorphisms (SNPs) and artefacts for Panel of Normal (PON) generation. The PON, along with the DogSD database (https://ngdc.cncb.ac.cn/idog/index.jsp) was used to clean artefacts and polymorphisms in the following variants calling. Variants calling was performed using Mutect2 with standard parameters. Called mutations were filtered by depth (> 30) and quality (> 20) and annotated with ANNOVAR using CanFam 3.1 from Ensembl Release 104 as reference genome. Short variants were classified as “UNKNOWN” if, as reported in ANNOVAR documentation, [a transcript maps to multiple locations, all as “coding transcripts”, but none has a complete ORF].

### 2.5 Tumour Mutational Burden (TMB) and Mutational Signatures

Tumour mutational burden (TMB) was calculated for each sample by dividing the total number of called variants (both coding and non‐coding) by the size in Mb of the haploid canine reference genome (CanFam3.1, 2.4 Gb) and reported as number of mutations/Mb ratio. Mutational signatures were extracted using the *Sigminer* R package. These signatures were then confronted against COSMIC signatures to extract valuable insight.

### 2.6 Somatic Copy Number Aberrations (SCNAs) and Structural Variants (SVs)

Copy Number Aberrations were accessed using ASCAT segmenter (version available in *EaCoN* R package). Segmented data underwent tumour purity and ploidy evaluation to estimate the major and minor allele number of copies. Additionally, segmented data were also used to run GISTIC2 peaks calculation. Both peaks and CNA regions were annotated using GTF file from UCSC and custom R script based on *rtracklayer* and *GenomicRanges* packages. Finally, structural variants (SV) were accessed using *Delly* and annotated with *Sansa*.

### 2.7 Statistical Analysis

Survival analysis was conducted using the *survival* and *survminer* R packages. Median TTP and LSS of the whole cohort were used as cutoff values to divide the dogs into “good” and “poor” responders. Survival curves were constructed using the Kaplan–Meier method. The Shapiro–Wilk test was employed to test TMB for normal distribution. The Student *t*‐test was employed to assess possible differences in TMB, the number of deleterious and tolerated missense variants, and the prevalence of truncating mutations between poor and good responders. The statistical significance threshold was set at *p* < 0.05.

## 3 Results

### 3.1 Study Population

**FIGURE 1:**
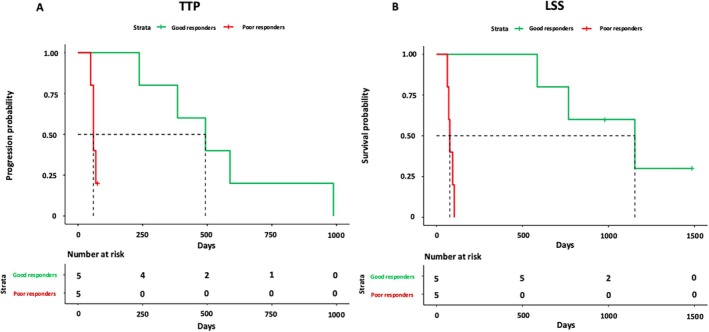
Kaplan–Meier (KM) curves for 10 dogs with DLBCL and treated with chemo‐immunotherapy according to clinical outcome. KM curves of TTP (A) and LSS (B) according to clinical outcome. Dogs classified as “poor responders” show both shorter TTP and LSS compared to those classified as “good” responders.

Ten cDLBCL cases with matched‐normal tissues were retrieved from the Canine Lymphoma Biobank [16]. Signalment and clinical data are reported in Table S1. The cohort consisted of two mixed breed dogs, two Dobermann, two Golden retrievers, and one each of West Highland White terrier, American Staffordshire, poodle, and German shepherd. Six (60%) dogs were males (of which one neutered), while 4 (40%) were females (of which three spayed). Median age was 7 years (range: 5–11 years) and median weight was 32.9 kg (range: 8.8–37.6 kg). Nine (90%) dogs had stage V disease, while 1 (10%) had stage IV disease. Four (40%) dogs showed clinical signs (substage b), while 6 (60%) did not (substage a). Six (60%) dogs had BM infiltration (median: 3.5%; range: 0.9%–47.4%), while 4 (40%) had PB infiltration (median: 1.7%; range: 0.9%–6.8%). Five (50%) dogs had elevated serum LDH activity, and 2 (20%) had been pre‐treated with steroids for 84 days and 9 days, respectively. While all dogs had received chemo‐immunotherapy, their treatment response varied significantly. The overall median TTP was 237 days, while the overall median LSS was 344 days (Figure S1). Based on these median values, the dogs of the cohort were categorised as either “poor” or “good” responders. Median TTP was 60 days (range: 49–76 days) for poor responders and 493 days (range: 237–988 days) for good responders (Figure 1A). Likewise, median LSS was 77 days (range: 63–103 days) for poor responders, while it was 1153 days (range: 585–1484 days) for good responders (Figure 1B).

### 3.2 Genome‐Wide Mutational Landscape of cDLBCL

**FIGURE 2:**
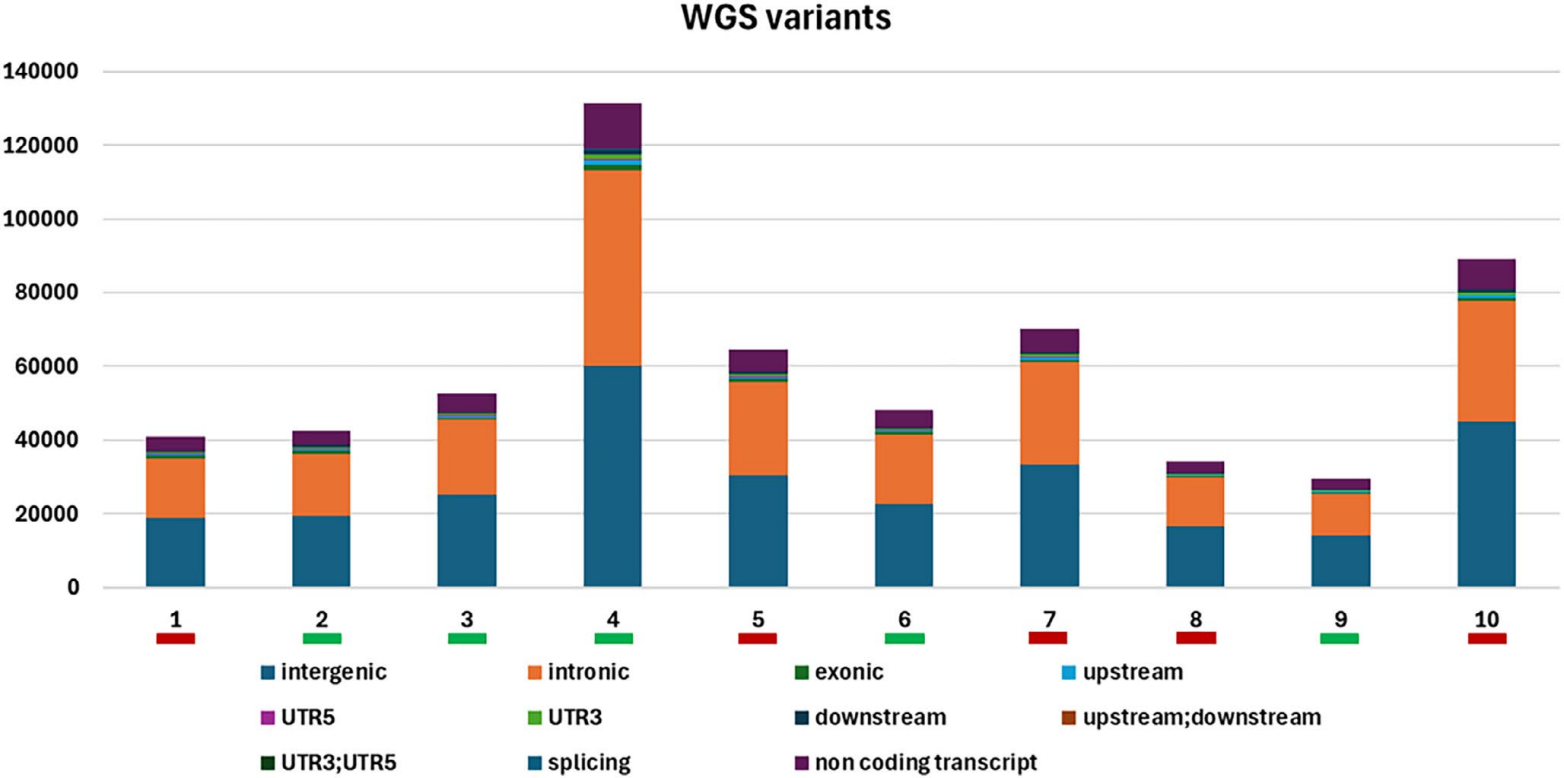
Variant distribution across 10 cDLBCL analysed by WGS. Distribution of somatic short variants (SNVs and indels) across 10 cDLBCLs. Each bar represents a single dog, while on the y‐axis is reported the total number of variants identified in each sample. “Poor” and “good” responders are indicated with red and green bars, respectively.

The median sequencing depth was 32.4× (range: 25–37×) for tumours and 25.1× (range: 13.5–28.5×) for normal samples. On average, 99.7% of reads aligned to the canine genome (range: 99.5%–99.8%), and there was a mean of 18% duplicate reads (range: 16.1%–22.0%). Collectively, the total number of SNVs and indels identified across tumours ranged from 29 368 to 131 378 (median: 50 259). Of these variants, 1.0% lay within coding regions (median: 1.2%; range: 0.7%–1.8%), while 99% represented non‐coding variants (median: 98.8%; range: 98.2%–99.2%) (Figure 2). The full list of the nucleotide variants is reported in Table S2.

### 3.3 cDLBCL Variants in Coding Regions

**FIGURE 3:**
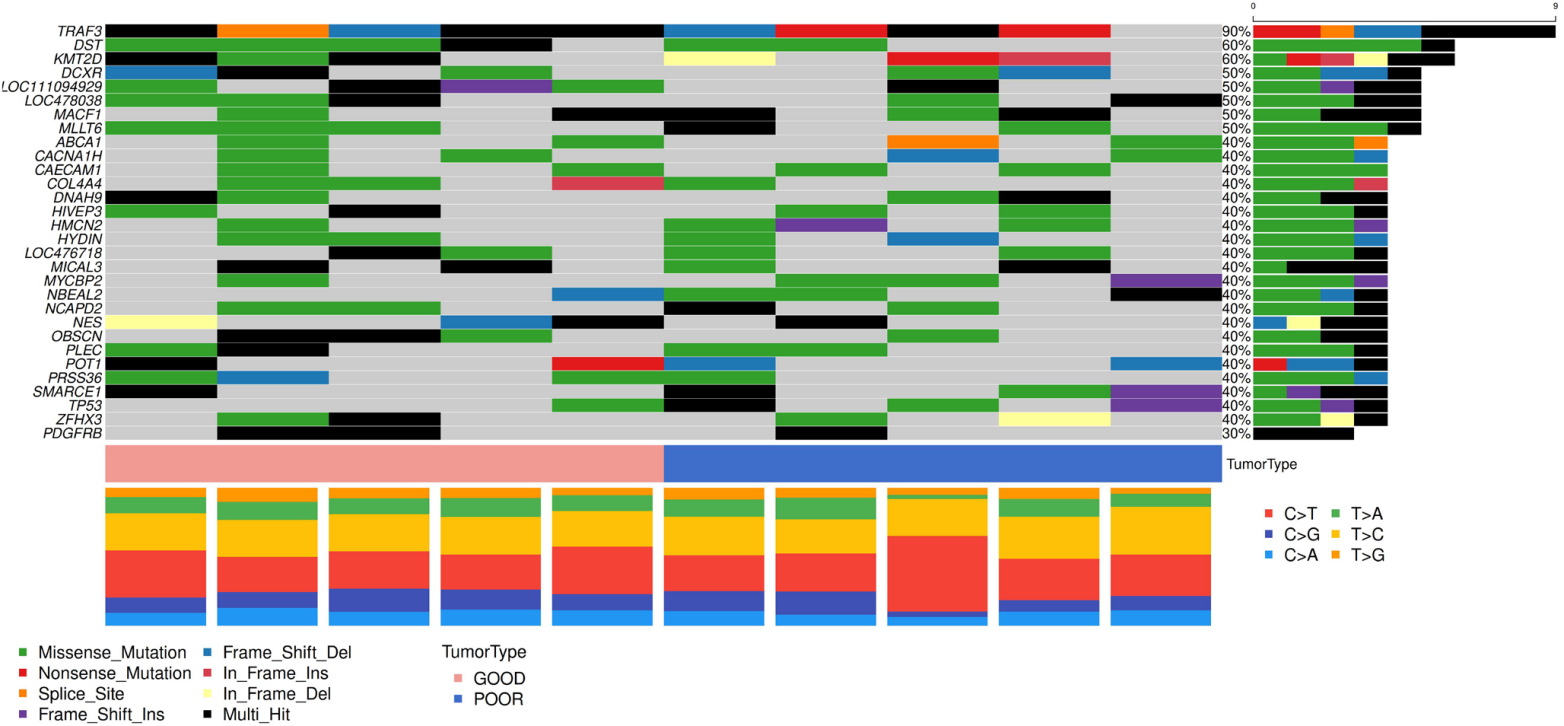
Oncoplot of genes carrying protein‐coding variants in cDLBCL. Top 30 mutated genes harbouring somatic protein‐coding SNVs and indels (upper panel) and distribution of nucleotide substitutions (lower panel) identified by WGS in 10 cDLBCLs. Genes are represented in descending order according to the frequency of mutation. Different mutation types are identified with different colours. Each column represents a single dog.

The median number of exonic variants per case was 653.5 (range: 368–1545), and a total of 5562 SNVs and 1279 indels in coding regions were identified. Among the SNVs, 1492 were synonymous (S), while 5536 were nonsynonymous (NS), with 256 determining the appearance of premature stop codons. This yielded a nonsynonymous‐to‐synonymous (NS:S) ratio of 3.7. Protein‐coding variants were retrieved in all dogs (Figure 3).

Missense mutations were the most frequent variants (53.9%), followed by frameshift mutations (13.2%). Inframe variants, nonsense mutations, stop‐loss, and start‐loss variants were 5%, 3.6%, 0.17%, and 0.16%, respectively. In addition, 2.7% of protein‐coding variants were classified as “unknown” (see Methods section).

Poor and good responders carried protein‐coding variants in 1910 and 2191 genes, respectively, and 1539 and 1820 genes were altered exclusively in poor and good responders, respectively. Among these, eight genes in the poor responder group (*TAF5L*, *CEP131*, *ABCA9*, *ETV1*, *PRAG1*, *LOC102155129*, *ARHGEF4* and *LOC102153036*) and nine genes in the good responder group (*ARFGEF3*, *SIPA1L3*, *COL12A1*, *ZBTB40*, *AMOTL1*, *MYO9A*, *DNAH17*, *ECEL1* and *IGSF9B*) were altered in at least three samples. *TRAF3* and *KMT2D* were altered in both groups.

By using the Sorting Intolerant From Tolerant (SIFT) tool, 1434 missense variants were predicted as deleterious, and 1685 as tolerated (Table S2). The number of deleterious and tolerated missense variants was not significantly different between poor and good responders (*p* = 0.6). Likewise, no statistically significant difference was retrieved in the number of truncating mutations (i.e., frameshift mutations, nonsense and start loss variants) between the two groups (*p* = 0.4).

### 3.4 cDLBCL Variants in Non‐Coding Genome

To broaden the spectrum of mutations in cDLBCL beyond coding regions, we focused on variants affecting splicing and the 3'‐and 5'‐untranslated regions (UTRs). Splicing variants were reported in all samples (median: 10.5; range: 6–30). The median number of 5'UTR and 3'UTR variants was 171.5 (range: 87–411) and 404 (range: 234–1118), respectively. The splicing and UTR variants impacted 4291 different genes. Of these, 688 had been reported in one previous canine B‐cell lymphoma exome study [7], and 13 were identified as recurrently mutated genes (≥ 5% of the samples) in cDLBCL (Table 1).

**TABLE 1:** Genes reporting splicing and UTRs variants that are mutated in at least 5% of cases of cDLBCL from WES studies.

Gene	N° mutated samples	Reported in COSMIC	Human lymphoid neoplasms	hDLBCL	cDLBCL (> 5%)
*LRRIQ1*	4	—	Yes	Yes	Yes
*FBXW7*	3	Yes	Yes	Yes	Yes
*HIVEP3*	2	—	Yes	Yes	Yes
*DIAPH2*	2	—	Yes	Yes	Yes
*TRAF3*	2	—	Yes	Yes	Yes
*ATXN1*	1	—	Yes	Yes	Yes
*KDM6A*	1	Yes	Yes	Yes	Yes
*ETV1*	1	Yes	Yes	Yes	Yes
*POT1*	1	Yes	Yes	Yes	Yes
*PHC3*	1	—	Yes	—	Yes
*PIK3CD*	1	—	Yes	Yes	Yes
*GBE1*	1	—	Yes	Yes	Yes
*RARA*	1	Yes	Yes	Yes	Yes

Additionally, 224 genes were listed in the Catalogue of Somatic Mutations in Cancer (COSMIC), while 3751 and 2463 genes were reported in human datasets of lymphoid neoplasms and DLBCL, respectively (Table S3). A total of 1814 genes were exclusively altered in poor responders, compared to 1843 that were mutated in good responders (Table S3).

The median number of intronic variants per case was 19 638 (range: 11406–53 023). In total, intronic variants were reported in 14 738 different genes. Among these, 625 were listed in the COSMIC database, while 12 560 and 8178 were reported in human lymphoid neoplasms and DLBCL datasets, respectively (Table S4). Thirty‐eight genes carrying intronic variants were recurrently mutated in cDLBCL (Table 2).

**TABLE 2:** Genes reporting intronic variants that are mutated in at least 5% of cases of cDLBCL from WES studies.

Gene	N° mutated samples	Reported in COSMIC	Human lymphoid neoplasms	hDLBCL	cDLBCL (> 5%)
*HIVEP3*	10	—	Yes	Yes	Yes
*LRRIQ1*	10	—	Yes	Yes	Yes
*FBXW7*	10	Yes	Yes	Yes	Yes
*VWF*	10	—	Yes	Yes	Yes
*ATXN1*	10	—	Yes	Yes	Yes
*ANKRD11*	10	—	Yes	Yes	Yes
*SYNE1*	9	—	Yes	Yes	Yes
*LRP1B*	9	Yes	Yes	Yes	Yes
*MEF2C*	9	—	Yes	Yes	Yes
*GBE1*	9	—	Yes	Yes	Yes
*TBL1XR1*	9	Yes	Yes	Yes	Yes
*SYNE2*	9	—	Yes	Yes	Yes
*MYT1L*	9	—	Yes	Yes	Yes
*ABCA13*	9	—	Yes	Yes	Yes
*ETV1*	8	Yes	Yes	Yes	Yes
*KIF21A*	8	—	Yes	Yes	Yes
*TTN*	8	—	Yes	Yes	Yes
*TRRAP*	8	Yes	Yes	Yes	Yes
*LAMA1*	8	—	Yes	Yes	Yes
*RARA*	8	Yes	Yes	Yes	Yes
*DIAPH2*	8	—	Yes	Yes	Yes
*PHC3*	8	—	Yes	—	Yes
*MAP3K14*	7	—	Yes	—	Yes
*SUZ12*	7	Yes	Yes	Yes	Yes
*TRAF3*	7	—	Yes	Yes	Yes
*FSIP2*	6	—	Yes	Yes	Yes
*THBS2*	5	—	Yes	—	Yes
*PLEC*	5	Yes	Yes	Yes	Yes
*PIK3CD*	5	—	Yes	Yes	Yes
*SETD2*	5	Yes	Yes	Yes	Yes
*KDM6A*	5	Yes	Yes	Yes	Yes
*CIC*	4	Yes	Yes	Yes	Yes
*POT1*	3	Yes	Yes	Yes	Yes
*FAM50A*	2	—	Yes	Yes	Yes
*DDX3X*	2	Yes	Yes	Yes	Yes
*EHD3*	2	—	Yes	Yes	Yes
*GADD45A*	1	—	—	—	Yes
*MYC*	1	Yes	Yes	Yes	Yes

Regarding oncologic outcome, 1927 genes were exclusively altered in poor responders, compared to 1965 that were mutated in good responders (Table S4).

Collectively, intergenic, downstream, and upstream variants ranged from 14 620 to 62 858 per sample, with a median of 24 815 variants. Variants in non‐coding transcripts (those affecting exons, introns, or splicing sites of non‐coding RNAs) were reported in all samples, with a median of 4682 variants (range: 2646–12 395) affecting 8045 different ncRNAs. To identify differentially mutated ncRNAs between poor and good responders, genes of uncertain function (LOC symbols) were not considered for further analyses. Ten ncRNAs were shared between poor and good responders, while 8 and 10 were exclusively mutated in poor and good responders, respectively. None of these was altered in more than one tumour from each group (Figure S2).

### 3.5 Tumour Mutational Burden and Mutational Signatures

The tumour mutational burden (TMB) ranged from 12.24 to 54.74 mutations per megabase (mean: 25.10; median: 20.94) and was normally distributed within the cohort. Samples #4 and #10 presented a higher TMB compared to the other tumours, and this may be due to the presence of several mutations affecting genes involved in various DNA repair mechanisms. Sample #4 presented frameshift mutations in *LIG1* and *MMS19*, involved in nucleotide excision repair (NER), and in *PNKP*, involved in the base excision repair (BER) mechanism. Sample #10 carried a frameshift mutation in *NEIL3*, involved in NER. No significant difference in TMB was retrieved between the poor and good responder groups.

**FIGURE 4:**
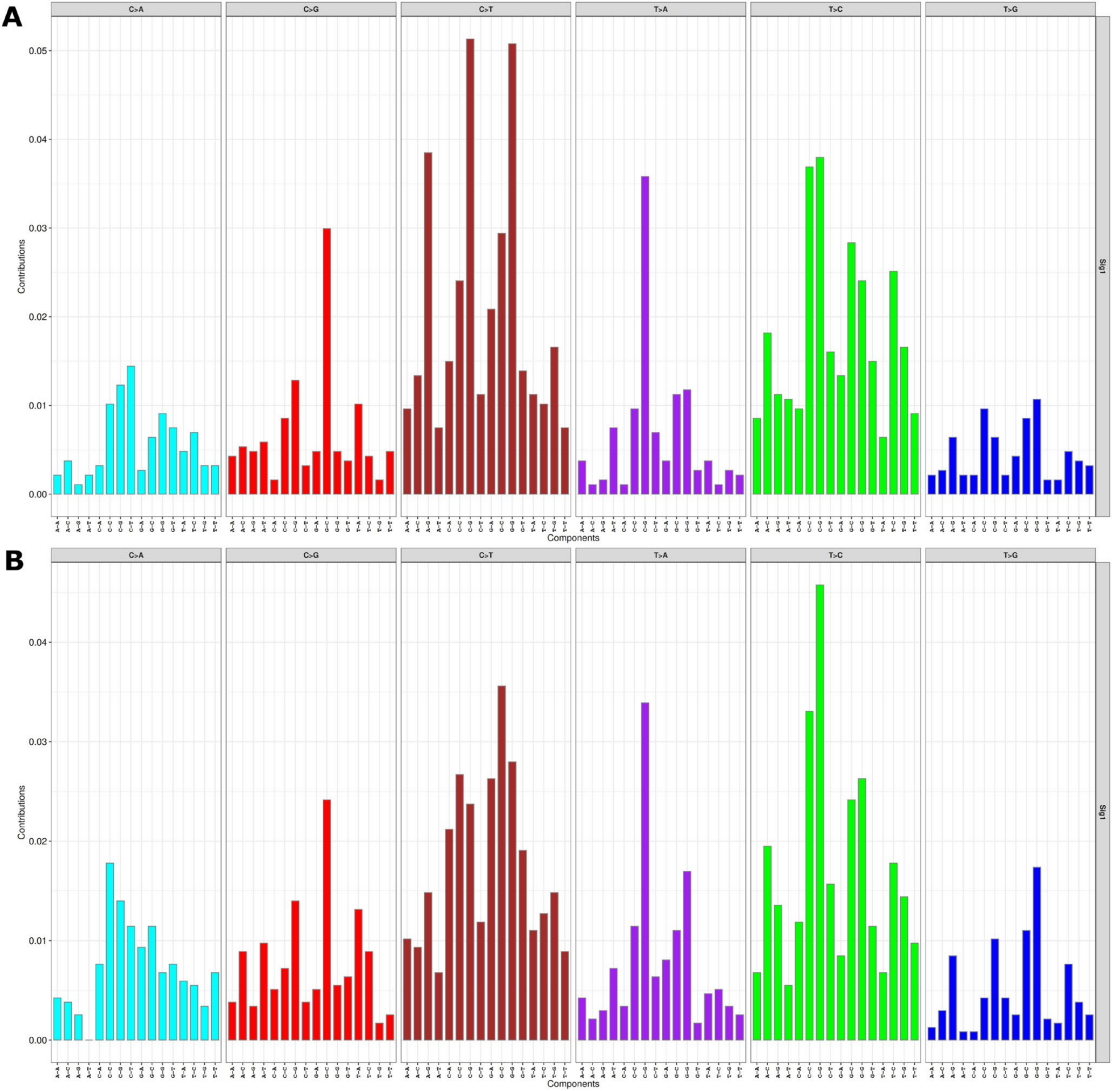
Mutational signatures analysis in cDLBCL. Mutational signatures were extracted using the Sigminer R package and confronted against COSMIC signatures. The plot shows the distribution of the six types of substitution (in 96 different trinucleotide contexts) defined by the pyrimidine as inferred from the non‐negative matrix factorisation algorithm. Both in poor (A) and good (B) responders, the analysis of single base substitution signatures (SBS) revealed that the signatures mainly contributing to mutation spectra corresponded to COSMIC SBS6 (defective DNA mismatch repair), SBS5 (clock‐like signature of unknown aetiology), SBS40c (of unknown aetiology) and SBS3 (defective homologous recombination DNA damage repair) in different proportions.

Mutational signatures analysis was performed using a Bayesian non‐negative matrix factorisation method to evaluate the trinucleotide context of somatic SNVs. Differential exposure analysis revealed no significant difference between the two groups. The analysis of single base substitutions (SBS) signatures revealed exposure to COSMIC signatures SBS6 (defective DNA mismatch repair), SBS5 (clock‐like signature of unknown aetiology), SBS40c (of unknown aetiology) and SBS3 (defective homologous recombination DNA damage repair) in different proportions (Figure 4, Table S5).

### 3.6 Somatic Copy Number Aberrations (SCNAs) and Structural Variants (SVs)

Absolute copy numbers were extracted from ASCAT segmentation files, revealing somatic copy number alterations (SCNAs) in nine tumours (median: 29; range: 9–217). Sample #5 was excluded from the analysis due to insufficient sequencing depth. The analysis identified 110 deletions (median: 7; range: 2–36), 309 amplifications (median: 16; range: 1–147), and 85 gain/loss events (median: 6; range: 3–39). Chromosome CFA31 exhibited the highest frequency of genomic rearrangements, comprising 25 amplifications, four deletions, and seven gain/loss events, while CFA24 demonstrated minimal alterations with only one deletion and two amplifications (Table S6).

Within amplified regions, several genes associated with transcriptional regulation were identified, including *POU1F1*, *USP16*, *PAXBP1*, *ZNF654*, *BACH1*, *CGGBP1*, and *VGLL3* (Figure S3). Conversely, genes located in deleted regions showed greater heterogeneity across tumours compared to those in amplified regions. Notable deletions were observed in *IGHM*, *MEF2C*, and *IGKC*, all of which are key components of the B‐cell receptor signalling pathway (Figure S4).

**FIGURE 5:**
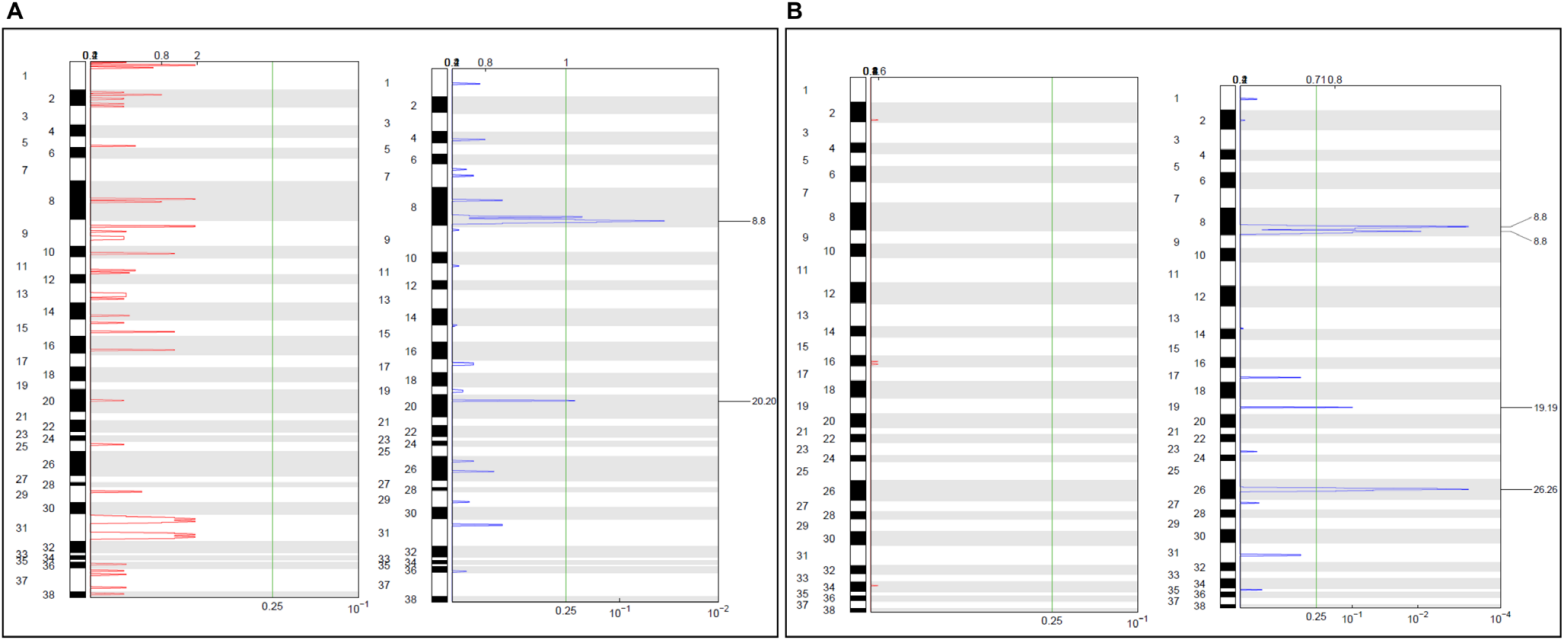
Recurrent somatic copy‐number aberrations (SCNAs) identified in cDLBCL. The picture shows statistically significant SCNAs peaks identified by GISTIC in poor (A) and good (B) responders. Amplification peaks are depicted in red, while deletion peaks in blue. Significance threshold is also indicated by a green line.

GISTIC analysis was employed to identify recurrent amplifications and deletions across the genome. In poor responders, two statistically significant deletion peaks were identified: one on CFA8 (73 027 410‐73 751 733; spanning 724 kb) and another on CFA20 (5 459 601‐12 452 795; spanning 7 Mb) (Figure 5A). Good responders exhibited four statistically significant deletion peaks: two on CFA8 (72 864 243‐73 365 210; spanning 501 kb, and 73 306 168‐74 292 901; spanning 987 kb), one on CFA19 (19 934 979‐25 959 251; spanning 6 Mb), and one on CFA26 (26 833 169‐27 628 477; spanning 795 kb) (Figure 5B). Notably, the CFA8 peaks detected in both groups showed partial overlap, corresponding to the canine IGH locus. Also, the peak on CFA26 corresponds to the IGL locus.

Structural variant analysis revealed extensive genomic alterations across all samples (median: 25 104; range: 21 715–36 142) (Table S7). The comprehensive analysis identified 68 255 inversions (median: 6703; range: 4399—10 280), 15 990 insertions (median: 1622; range: 1378–1742), and 6447 duplications (median: 656.5; range: 405–854).

## 4 Discussion

In the last decades, the application of genome sequencing technologies to cancer research has revolutionised both diagnostics and therapeutics. These advancements have not only reinforced the understanding that cancer is fundamentally a genetic disease but have also uncovered the existence of multiple molecular subgroups within the same tumour histotype. Each subgroup may have a distinct clinical course, prognosis, and response to therapy, highlighting the complexity and heterogeneity of cancer.

DLBCL represents the most common histotype among all hematologic malignancies in humans and dogs, and several comparative studies have highlighted both similarities and differences between the two species [18, 19]. In humans, comprehensive analysis of data from hundreds of DLBCL exomes and genomes has identified numerous distinct molecular subtypes, each characterised by specific genetic aberrations and associated with varying prognosis [20, 21].

In the present study, 10 dogs diagnosed with DLBCL, exhibiting similar clinico‐pathological variables but significantly different clinical outcomes, underwent WGS to determine whether these differences could be attributed to distinct molecular characteristics.

Considering protein‐coding variants, 8 and 9 genes were identified as being exclusively altered in poor and good responders, respectively. Among the genes specific to poor responders, *TAF5L* encodes a protein that is a component of the P300/CBP‐associated factor (PCAF) histone acetyltransferase complex. It has been demonstrated that TAF5L/TAF6L act as epigenetic regulators, transcriptionally activating the *MYC* regulatory network, which is crucial for maintaining self‐renewal in mouse embryonic stem cells [22]. *CEP131* encodes a protein involved in many cellular processes, including protein localisation to the centrosome. Different studies have reported that *CEP131* functions as an oncogene, promoting carcinogenesis in breast cancer [23] and tumour progression in colon cancer [24]. Also, *CEP131* overexpression has been reported as a predictor of poor prognosis in hepatocellular carcinoma [25] and neuroblastoma [26]. In our cohort, we reported three missense variants in *TAF5L* and two missense variants and an in‐frame variant in *CEP131*. Since the impact of non‐truncating variants is challenging to predict, it will be essential to investigate whether these variants lead to alterations in gene and protein expression. This will help determine if they may play a role in contributing to the worse clinical outcome observed in these dogs. *ABCA9* encodes a gene belonging to the superfamily of ATP‐binding cassette (ABC) transporters. One study reported that in a subset of hepatocellular carcinoma human patients, *ABCA8* and *ABCA9* downregulation was significantly associated with shorter survival time [27]. In our cohort, three dogs in the poor responders group carried *ABCA9* mutations, specifically two frameshift mutations and one nonsense variant, which represent loss‐of‐function mutations likely associated with reduced protein expression. Additionally, *ETV1* is the only gene listed in the COSMIC database, and chromosomal translocations involving this gene have been implicated as causative factors in Ewing Sarcoma [28] and prostate cancer [29].

Among the genes found exclusively in the good responder group, *COL12A1* upregulation has been identified as a predictor of poor prognosis in human patients with pancreatic adenocarcinoma [30] and gastric cancer [31]. Meanwhile, *AMOTL1* has been reported as recurrently mutated in splenic marginal zone lymphoma [32].

To expand the spectrum of cDLBCL mutations beyond coding regions, we first interrogated splicing and UTR variants that could affect proper gene expression in different ways. In our cohort, 13 genes previously identified as recurrently mutated in cDLBCL [7] exhibited splicing and/or 5′‐and 3′UTR variants. In the poor responder group, *FBXW7* exhibited a splicing variant and two 3′UTR variants, while *DIAPH2* had both a 3′‐and a 5′UTR variant. Additionally, *KDM6A* contained two 3′UTR variants in the same dog (sample #8). In the good responder group, *TRAF3* carried two 3′UTR variants in sample #2 and a splicing variant in sample #4. Moreover, *RARA* presented a 5′UTR variant, while *POT1*, *PIK3CD*, and *GBE1* presented a 3′UTR variant each. Biologically, splicing variants can disrupt an existing splice site or introduce a new one, leading to various outcomes such as exon skipping, intron retention, or the utilisation of alternative splicing sites located within introns [33]. UTR variants can influence gene expression through different mechanisms, including alternative splicing, reduced translational efficiency, and altered interactions with proteins and miRNAs [34]. Although these results suggest a potential role for these types of genetic aberrations in the pathogenesis and prognosis of cDLBCL, further genetic screening involving a larger number of cases, along with in vitro functional studies, is necessary to validate this hypothesis.

Non‐coding mutations can contribute to tumorigenesis through the alteration of *cis*‐regulatory elements, including promoters and distal enhancers, the disruption of chromatin 3D structure, and altered function of regulatory non‐coding RNAs (ncRNAs) [35]. In humans, the analysis of 2658 cancer genomes across 38 different tumour types identified somatic non‐coding driver mutations [36]. In DLBCL, hypermutation of active super‐enhancers linked to known proto‐oncogenes has been identified as a novel mutational mechanism involved in the pathogenesis of this tumour [37].

In our cohort, we identified variants in intronic, intergenic, and ncRNA regions. However, the limited number of cases prevented us from identifying recurrent mutations that may affect regulatory regions or from distinguishing driver events from passenger mutations. Comparative studies have shown that many regulatory elements are located within evolutionarily conserved regions known as conserved non‐coding elements (CNEs). Disruption of these CNEs has been implicated in developmental disorders and cancer [38].

TMB and mutational signatures were also examined. Although TMB did not differ significantly between poor and good responders, two samples (sample #4 and sample #10) exhibited markedly higher TMB compared to the others. This is likely due to the presence of loss‐of‐function mutations in genes involved in NER and BER pathways. In human oncology, TMB has emerged as a valuable biomarker for identifying patients who may benefit from immunotherapy [39]. A possible explanation for the differing therapeutic responses observed in these two cases is that, although patient #4 had a high number of retrieved variants, their mutational load in coding regions was actually lower than that of patient #10. This discrepancy arises because, in the present study, TMB was calculated based on both coding and non‐coding variants.

The assessment of mutational signatures did not show substantial differences between poor and good responders. The signatures that predominantly contributed to the mutation spectra were identified as COSMIC SBS6 (associated with defective DNA mismatch repair), SBS5 (a clock‐like signature of unknown aetiology), SBS40c (also of unknown aetiology) and SBS3 (linked to defective homologous recombination DNA damage repair). This contrasts with the findings of Giannuzzi et al. [7], where the analysis of 77 cDLBCL through WES identified SBS1 (age‐related spontaneous deamination of 5′‐methylcytosine) as the predominant signature. A lack of correspondence in mutational signatures between WGS and WES has been previously noted in canine osteosarcoma by Gardner et al. [40], who suggested that this discrepancy may be due to the higher sequencing depth typically achieved with WES.

SCNAs represent a common event in cancer, and recurrent alterations associated with specific histotypes have been identified [41]. In dogs, SCNAs and SVs have been described in osteosarcoma [40, 42], melanoma [43, 44], hemangiosarcoma [45] and mammary tumours [46]. In our cohort, a recurrent deletion peak on CFA20 was observed in poor responders, while two recurrent deletion peaks on CFA19 and CFA26 were identified in good responders. Additionally, recurrent deletions affecting the sub‐telomeric region of CFA8, which corresponds to the canine IGH locus [47], were reported in both groups, likely due to V(D)J genes rearrangement. Among the 56 genes located within the CFA20 deletion, germline variants in *MBD4*, a gene encoding for a mismatch‐specific DNA N‐glycosylase, have been reported as a predisposing factor for acute myeloid leukaemia [47]. Additionally, *MBD4* deficiency has been identified as a predictive marker for response to immune checkpoint inhibitors in uveal melanoma [48]. *VGLL4* loss was correlated with the suppression of PDL‐1 expression [49]. Inactivating mutations in the tumour suppressor gene *VHL* have been associated with several diseases, including pheochromocytoma, erythrocytosis, renal cell carcinoma, and cerebellar hemangioblastoma [50]. Additionally, *FANCD2* deletions are implicated in Fanconi anaemia, a genetic disease characterised by chromosomal instability and defective DNA repair [51].

In conclusion, our study provides a comprehensive view of the genomic landscape of cDLBCL at a genome‐wide resolution. Despite the main limitations being the small sample size and relatively low coverage, we extended the analysis beyond coding regions, uncovering a broader spectrum of potentially pathogenic variants in genes already known to be recurrently mutated in cDLBCL. Additionally, we identified novel candidate genes that may play a role in the disease's pathogenesis. Further studies with larger cohorts are needed to validate these findings.

## Conflicts of Interest

The authors declare no conflicts of interest.

## Supporting information


**FIGURE S1.** Median time to progression (TTP) and lymphoma‐specific survival (LSS) of the whole cohort. The Kaplan–Meier (KM) curves present median TTP (left) and LSS (right) of the whole cohort of 10 dogs with cDLBCL.

**FIGURE S2.** Oncoplot of non‐coding (nc) transcripts mutated in 10 cDLBCL. Genes specific for ncRNAs (excluding LOCs) are reported in descending order of mutation frequency. Each column represents a single dog. The top 10 genes were retrieved in both groups, while the remaining were reported in a single case from poor or good responders (indicated with the blue and the pink bar at the bottom, respectively) each. The distribution of single nucleotide substitutions is also depicted.

**FIGURE S3.** Oncoplot of amplified genes. Amplified genes are reported in descending order of frequency of amplification. Poor and good responders are indicated as purple and green bars at the bottom, respectively. For sample #5 it was not possible to extract copy numbers because of insufficient sequencing depth.

**FIGURE S4.** Oncoplot of deleted genes. Deleted genes are reported in descending order of frequency of deletion. Poor and good responders are indicated as purple and green bars at the bottom, respectively. For sample #5 it was not possible to extract copy numbers because of insufficient sequencing depth.

**TABLE S1.** Clinical details for 10 dogs with DLBCL sequenced in this study.

**TABLE S2.** Short somatic variants (SNVs and indels) identified in 10 cDLBCL through WGS.

**TABLE S3.** Genes reporting splicing and UTR variants in 10 dogs with cDLBCL. Genes exclusively altered in poor or good responders are highlighted in orange and green, respectively.

**TABLE S4.** Genes reporting intronic variants in 10 dogs with cDLBCL. Genes exclusively altered in poor or good responders are highlighted in orange and green, respectively.

**TABLE S5.** Single base substitutions (SBS) signatures similarity against COSMIC signatures.

**TABLE S6.** Absolute copy numbers extracted from ASCAT segmentation files. For sample#5 was not possible to retrieve CNAs because of insufficient sequencing depth.

**TABLE S7.** Structural variants (SVs) found in 10 dogs with cDLBCL.

## Data Availability

The data that support the findings will be available in BioProject at https://www.ncbi.nlm.nih.gov/bioproject/ following an embargo from the date of publication.
