# Supplementary figures and images for: Expanding the Spectrum of Canine Diffuse Large B‐Cell Lymphoma Genetic Aberrations Through Whole Genome Sequencing Analysis
Source: Vet Comp Oncol. 2025 May 4;23(3):346–57. doi: 10.1111/vco.13059 (PMC12378081; doi:10.1111/vco.13059)

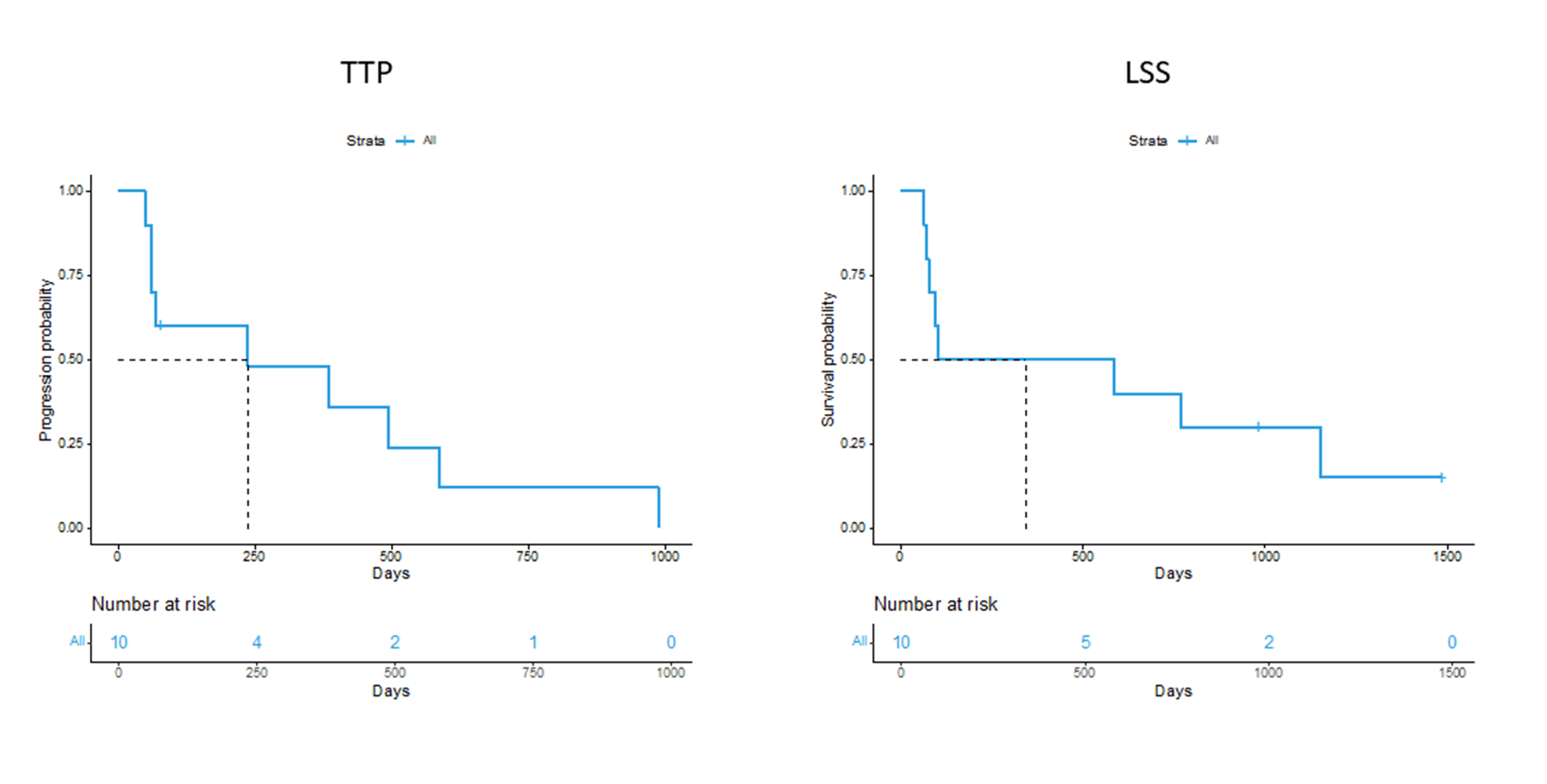

Supplement: Supplementary file 1 — FIGURE S1. Median time to progression (TTP) and lymphoma‐specific survival (LSS) of the whole cohort. The Kaplan–Meier (KM) curves present median TTP (left) and LSS (right) of the whole cohort of 10 dogs with cDLBCL. [file VCO-23-346-s005.tif]

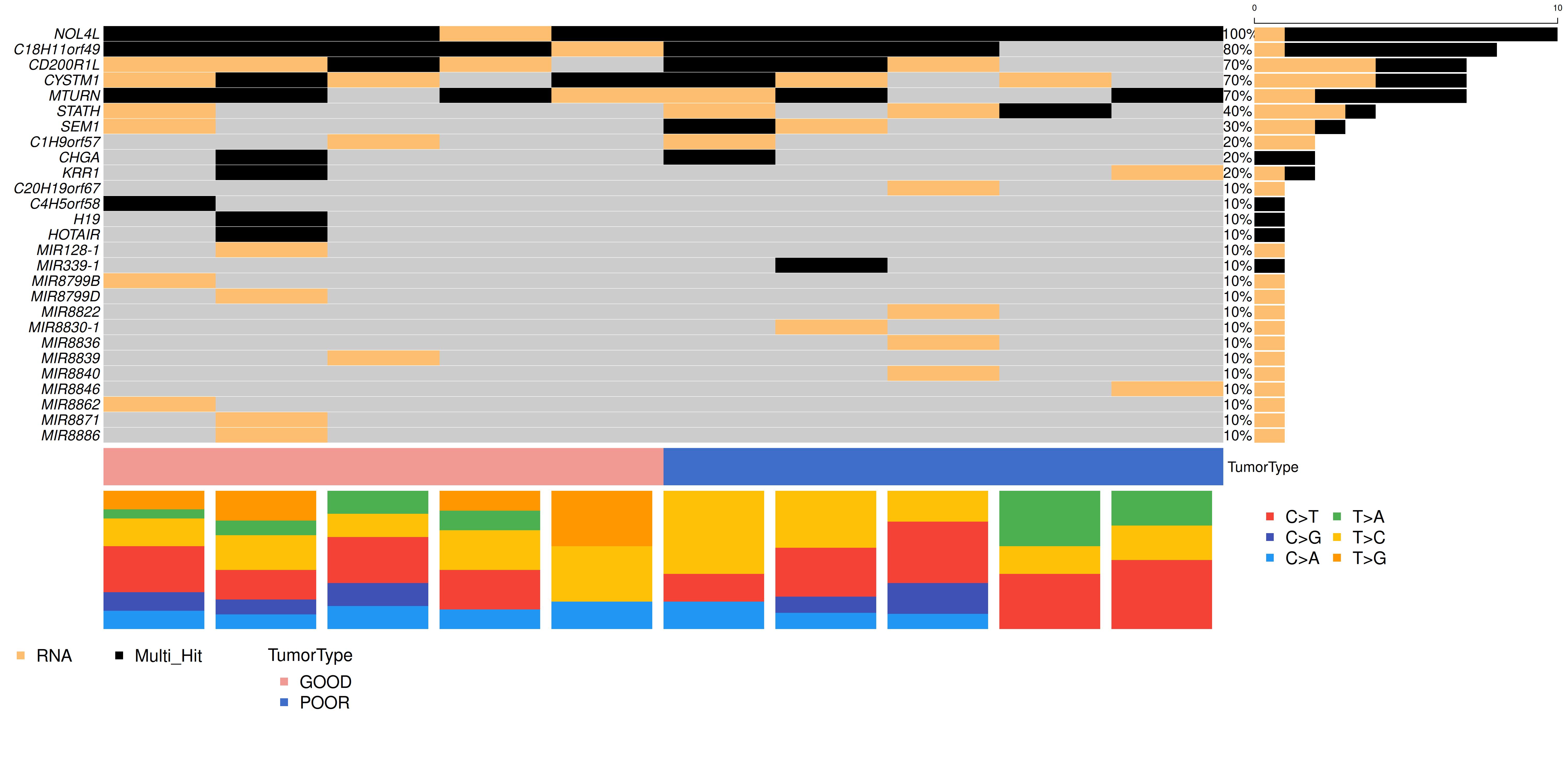

Supplement: Supplementary file 2 — FIGURE S2. Oncoplot of non‐coding (nc) transcripts mutated in 10 cDLBCL. Genes specific for ncRNAs (excluding LOCs) are reported in descending order of mutation frequency. Each column represents a single dog. The top 10 genes were retrieved in both groups, while the remaining were reported in a single case from poor or good responders (indicated with the blue and the pink bar at the bottom, respectively) each. The distribution of single nucleotide substitutions is also depicted. [file VCO-23-346-s007.jpeg]

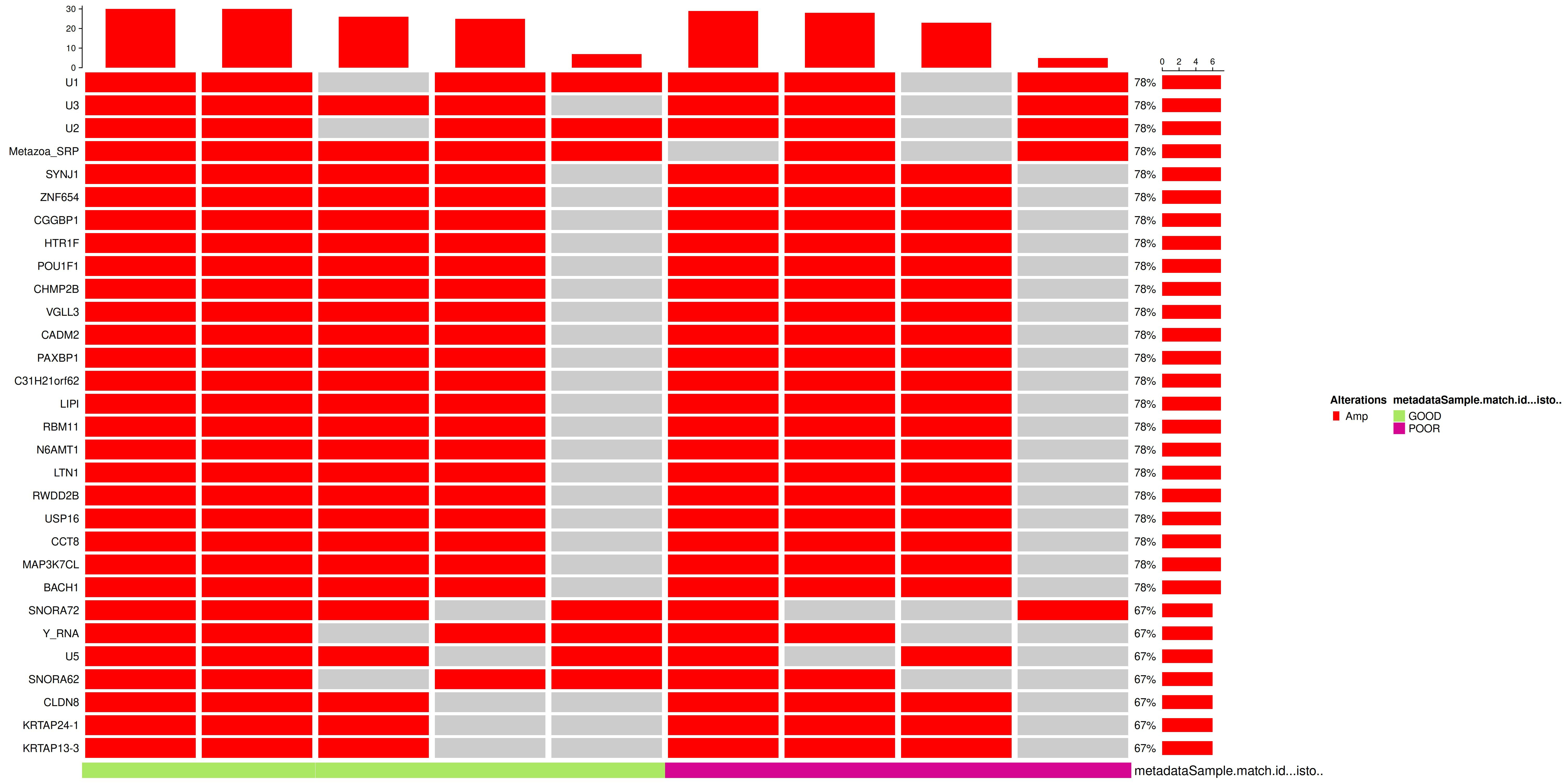

Supplement: Supplementary file 3 — FIGURE S3. Oncoplot of amplified genes. Amplified genes are reported in descending order of frequency of amplification. Poor and good responders are indicated as purple and green bars at the bottom, respectively. For sample #5 it was not possible to extract copy numbers because of insufficient sequencing depth. [file VCO-23-346-s002.jpeg]

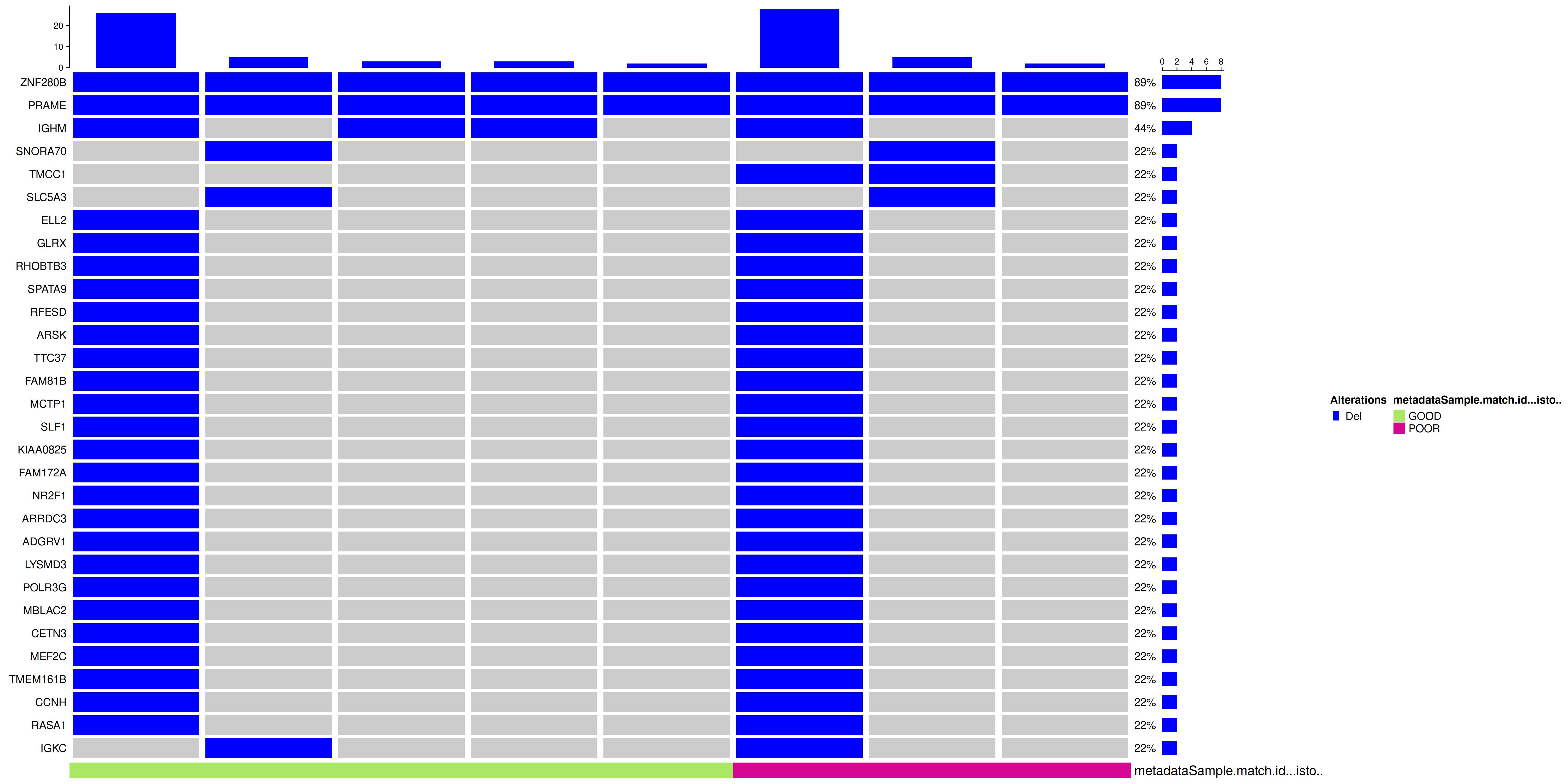

Supplement: Supplementary file 4 — FIGURE S4. Oncoplot of deleted genes. Deleted genes are reported in descending order of frequency of deletion. Poor and good responders are indicated as purple and green bars at the bottom, respectively. For sample #5 it was not possible to extract copy numbers because of insufficient sequencing depth. [file VCO-23-346-s011.jpeg]
